# Is Dichotomization into Regular versus Irregular Dental Attenders Valid? A Qualitative Analysis

**DOI:** 10.1177/23800844221118515

**Published:** 2022-08-29

**Authors:** M.M. van der Zande, C.E. Exley, R. Freeman, C. Thetford, R.V. Harris

**Affiliations:** 1 Department of Public Health, Policy & Systems, Institute of Population Health, University of Liverpool, Liverpool, UK; 2 Population Health Sciences Institute, Newcastle University, Newcastle, UK; 3 School of Dentistry, University of Dundee, Dundee, UK; 4 School of Nursing, University of Central Lancashire, Preston, UK

**Keywords:** health service utilization, inequalities, periodontal risk, oral health behavior, preventive health care, compliance

## Abstract

**Aims:**

To discover whether dental visiting behavior can be understood as a dichotomy of planned versus problem based, or whether there were a range of different types of understanding and patient behavior, recognizable as patterns of dental visiting behavior.

**Methods:**

Secondary analysis drawing on 2 qualitative studies of patients’ accounts of dental attendance and oral health, with 1) opportunistic interviews with people attending urgent dental care services (*n* = 43; including 19 with follow-up) and 2) home-based, in-depth interviews with people attending a dental practice with a mixture of improved or deteriorated/poor periodontal health (*n* = 25).

**Results:**

Four distinguishable patterns of dental visiting were identified in patients’ accounts: Accepting and Active Monitoring, as well as Ambivalent and Active Problem-based dental visiting behavior. Individuals’ patterns were relatively stable over time but could shift at turning points. Accepting Monitors were characterized as accepting dentists’ recommendations and dental practice policies relating to oral health and visits, whereas Active Monitors were more independent in judging how often to attend for preventive appointments, while still valuing anticipatory care. Ambivalent Problem–based visitors placed a relatively low value on anticipatory care for oral health maintenance and drifted into lapsed attendance, in part because of service-related factors. This contrasted with Active Problem–based visitors, for whom using services only in an emergency was a conscious decision, with low value placed on anticipatory care.

**Conclusion:**

This article demonstrates the dynamic nature of patterns of dental visiting where the dental system itself is partly instrumental in shaping patterns of utilization in an ecological way. Thus, service-related factors tend to combine with patients’ behavior in expanding inequalities. This illuminates the reasons why risk-based recalls are challenging to implement as a dental policy.

**Knowledge Transfer Statement:**

The results of this analysis can be used by clinicians and policymakers to inform policy around supporting uptake of preventive health care visits, contributing in particular to understanding how risk-based preventive visiting policies may be better adapted to patients’ understanding of the purpose of visits, taking into account that this is in part shaped by service-related factors in an ecological way, arising from patients’ and dental teams’ expectations.

## Introduction

Regular check-ups are recommended in dentistry for several reasons: first as a screening (Armstrong and Eborall 2012) for oral diseases so that an early diagnosis can be made such as for dental caries, periodontal disease, and oral cancer (Fee et al. 2020). In addition, check-ups give an opportunity for early preventive interventions to limit disease progression (e.g., by applying fluoride varnish) (Fee et al. 2020) and providing advice on behaviors such as the technique and timing of toothbrushing (Fee et al. 2020; Soldani et al. 2018). Patients are more likely to comply with such advice when dental visits are maintained over time and where patient–dentist relationships have developed, which is an important reason why an ongoing program of visits is advantageous (Hill et al. 2013).

Dental check-ups are a type of anticipatory care, creating a point of contact between patients and a dental team that allows for early intervention. Preventive health checks are a cornerstone of current UK health policy aimed at improving health equity (Lorant et al. 2002), so the principle of dental check-up visits is now somewhat mirrored in the relatively new health checks undertaken in National Health Service (NHS) general medical practice (Dryden et al. 2012). However, there are key differences between the general medical practice system and the general dental practice system, since although simple clinical examinations represent 15% of all spending in primary dental care (Fee et al. 2020), the majority of primary health care provision is focused on providing curative care in response to patients’ needs (OECD 2013).

A large body of scientific evidence shows inequalities in the use of preventive health care (Lorant et al. 2002; Armstrong and Eborall 2012; Dryden et al. 2012), with people from less advantaged backgrounds less likely to use health services preventively (Dryden et al. 2012). These inequalities are larger for preventive medicine than for curative health care (Lorant et al. 2002). We see this particularly clearly in dentistry, with a social patterning of preventive dental visiting that is lower among people from the most disadvantaged backgrounds (Sanders et al. 2006), with inequalities larger than for other types of preventive health care visiting (Kino et al. 2019). These inequalities are rooted in social class (Donaldson et al. 2008), socioeconomic conditions (Guarnizo-Herreño et al. 2021), and health care system characteristics (Kino et al. 2017) and linked to long-term dental visiting via intergenerational effects (Broadbent et al. 2016) and health beliefs (Donaldson et al. 2008; Broadbent et al. 2016).

Overall, 61% of UK adults usually visit a dentist for a check-up (Hill et al. 2013). Similarly, in 27 European countries, an average 63% of adults report visiting a dentist for a check-up in the past 12 months (Kino et al. 2017), and globally, an average 54% of children and adults visit a dentist regularly or preventatively (Reda et al. 2018). It is, however, relatively difficult to define “regular/preventive” dental visiting behavior apart from this generally being the inverse of “problem-based” dental visiting, sometimes with the addition of an intermediate category of “occasional” dental visiting between the 2 poles of the dental visiting behavior spectrum (McGrath and Bedi 2001; Reda et al. 2018).

Problem-based visiting is usually defined as visiting only when experiencing acute dental pain or dental problems (Currie et al. 2021). However, this categorization is problematic and does not reflect variations in dental visiting adequately, first because more recent studies have shown that dental visiting over the life course often involves a mix of periods of regular and irregular dental visiting. Long-term or life course dental visiting patterns show a clearer association with oral health than the dichotomous (regular/not-regular) approach (Thomson et al. 2010; Aldossary et al. 2015). Secondly, the concept of “regular” dental visiting combines the elements of frequency of dental visits, such as a dental visit for any reason in the past 12 months (McGrath and Bedi 2001), with reasons for dental visits, such as attending for a check-up (Hill et al. 2013). Regular visiting is thus sometimes taken as the counterpart of “irregular” dental visiting—not having had a visit in the past 12 months (McGrath and Bedi 2001) and sometimes of visiting the dentist for a dental problem (Donaldson et al. 2008; Hill et al. 2013; Currie et al. 2021), which is conceptually imprecise as well as confusing.

The third reason the categorization of “regular” dental visiting is contested is that the optimal frequency of dental visits for planned care has been debated (Sheiham 1977; Kay 1999), but this has not led to reconsideration of conventions in how dental visiting is categorized. Current guidance recommends risk-based frequency of dental check-ups—with intervals tailored according to the individuals’ oral health risk (NICE 2004). These risk-based intervals should be determined following a full assessment of the patient’s oral disease and risk and agreed on jointly by the dentist and patient (NICE 2004). Recent evidence shows no significant difference in oral health between 6 month and risk-based intervals and between 6 month, 24 month, and risk-based intervals for patients with low risk (Clarkson et al. 2020; Fee et al. 2020). Furthermore, 24 month intervals can reduce costs to both health services and patients (Clarkson et al. 2020). However, patients’ preferences are often for shorter and standardized time intervals (Clarkson et al. 2020). Moreover, although dentists’ practices are changing toward risk-based intervals, standard intervals are still used widely (Mettes et al. 2006). Either way, impacts on inequalities are a key consideration, and more research is needed to understand why NICE guidance recommended extending periods between check-ups for low-risk patients has seen relatively slow adoption by the system.

Dental visiting behavior appears to be shaped by how patients understand the purpose of the visit and may be shaped by the expectations and preferences held by the dental practitioner, yet a clear understanding of patients’ accounts of dental visiting behavior is lacking, particularly of problem-based dental visiting (Currie et al. 2021). We aimed to first discover whether dental visiting behavior can be understood as a dichotomy of planned versus problem based or whether there were a range of different types of understanding and patient behavior, recognizable as patterns of dental visiting behavior that could inform defining a typology of dental visiting behavior. Second, we aimed to identify changes that may occur in these patterns over time and any symbiosis with dental service factors. This article thus presents patients’ accounts of dental visiting, aiming to understand dental visiting behavior types, to inform both the terminology used in this field as well as future research and health policy relating to extending recall intervals between dental check-ups for patients at low risk of oral disease.

## Methods

The article presents secondary analysis drawing on 2 qualitative studies on patients’ accounts of dental attendance and oral health behaviors. In this section, we describe the data, followed by discussion of the methodological approach adopted in this study, and selection of data and analytic approaches.

Data from study 1 and study 2 consisted of interviews, both eliciting narratives where the participants reflected on their lifetime experience of dental visits. Study 1 involved participants attending because they had a problem. Study 2 was included to complement this with a patient population with a more ongoing pattern of dental practice visiting, involving interviews conducted in participants’ homes, providing more depth than was possible in interviews conducted opportunistically in urgent care settings (topic guides in the Appendix).

Study 1 involved an ethnographic study (van der Zande et al. 2021), part of the RETURN program aiming to understand and reduce inequalities in uptake of dental visits among people attending urgent dental care who had not attended dental care for some time. Study 1 was conducted in 2018–2019, in 6 dental care settings: a dental hospital providing walk-in urgent dental care, 2 dental practices providing in-hours urgent dental care, and 3 clinics providing out-of-hours urgent dental care. All were in an urban setting in northwest England, which experiences a considerable burden of ill health and substantial use of urgent dental services. Ninety-seven participants were interviewed before or after their appointment in the urgent dental care setting, after receiving information about the study and providing written consent. Nineteen of the original participants also participated in follow-up interviews (all participants who were consented and were contactable were approached), which were conducted around 6 months after urgent care by telephone. Men and younger people are usually overrepresented among urgent care attenders (Worsley et al. 2016) compared to the general population, and the sample reflected this. Interviews explored barriers to dental attendance and participants’ strategies to overcome these. Ethical approval was obtained from the Health Research Authority North-East, Tyne and Wear South (18/NE/0061, IRAS ID 240819).

Wanting to include participants with diverse socioeconomic circumstances and knowing that patient compliance and oral hygiene behaviors follow a similar social patterning to dental visiting (Sanders et al. 2006), we used study 2. Study 2 involved biographical interviews with 25 participants all recruited as patients of a single dental practice in an area with high levels of deprivation in an urban setting in northwest England, conducted in 2012. This study’s purposive sample was based on clinical record findings contrasting patients whose periodontal health had remained stable or worsened over time with patients whose periodontal health had improved. Older groups were overrepresented in this sample compared to the general population, which could be because periodontal disease risks increase with age (López et al. 2017). Participants with periodontal health problems were given information about the study by the dental staff at their dental practice or by the researcher who was present in the dental practice, and they provided written consent prior to the interviews. Ethical approval was obtained from the Health Research Authority North-West, Preston (08/H1016/6).

### Methodological Approach

Secondary analysis of qualitative data enables combined analysis of research questions that could not be answered with primary research producing 1 data set and can be used in research where gathering new data would not be possible (e.g., in historical research) or would pose an unnecessary burden on participants (Hammersley 2010). However, it brings particular challenges, relating to in-depth understanding of the context in which research data were gathered and fit of the research question to the data gathered (Hammersley 2010; Irwin 2013). Although both of these challenges are not limited to secondary analysis, they are particularly salient therein (Hammersley 2010).

Data gathered from interviews in both study 1 and study 2 underwent primary analyses (van der Zande et al. 2021) and secondary analysis reported here. The primary analyses included ample data on how dental attendance is approached by the participants, which was beyond the primary analyses’ aims. This became the focus of the secondary analysis. The data complemented each other by including both participants who had a period without dental attendance (study 1) and participants with recent attendance for preventive maintenance in 1 dental practice (study 2). We believe therefore that these primary studies fit the aims of this secondary analysis, and given that data were already available, primary data collection would place an unnecessary burden on participants and resources.

The context in which the research data were produced was taken into account in this study by having the researchers who conducted the primary studies involved in the secondary analysis, which proved valuable during analysis. Data for study 1 were gathered by MMZ, a medical sociologist, and in the dental hospital setting in part by RVH, a public health researcher, and a small proportion by a research assistant with a background in psychology; data for study 2 were gathered by CT, a health services researcher. The analytic team also included CEE, a medical sociologist, and RF, a dental public health academic. The researchers of both studies had gained some distance from the primary studies through the passing of time when secondary analysis was conducted (Irwin 2013).

### Selection of Data and Analysis

From the transcripts available in study 1, only those of interviews longer than 15 min were included in this analysis, as well as of all participants who participated in follow-up interviews, to make sure accounts provided enough depth (*n* = 43). Follow-up interview transcripts were also included (*n* = 19). From study 2, all transcripts available in the data set were included (*n* = 25).

Data were analyzed using thematic analysis (Braun and Clarke 2006), facilitated by NVivo version 12 (QSR International Pty. Ltd.). Initial analysis centered on dental attendance patterns over time, focusing on turning points in dental attendance. In addition to thematic analysis, diagramming (Buckley and Waring 2013) was used in this phase as a visual aid to map personal, dental care service and oral health–related factors that influenced dental attendance over the life course, as well as to understand differences among dental attendance patterns. Elements of dental attendance and behaviors shaped by dental care services emerged as crucial themes, which address a gap in the literature on dental attendance. We subsequently returned to the data with a focused analysis on the constitutive elements of dental attendance, motives for dental attendance, and behaviors and motives shaped by dental care services. Patterns of dental attendance in participants’ accounts were then compared within and across different patterns, arriving at a typology of dental attendance patterns.

Data analysis was conducted by MMZ, and emerging interpretations were discussed regularly among the authors. Participants’ names and any other identifiable details have been changed to ensure confidentiality.

## Results

Participants’ characteristics are detailed in the Table. Eighty-seven interviews (including follow-ups) with 68 participants were included across the 2 studies, 38 of whom were male and 30 female, ranging in age from 19 to 75 y. All personal names are pseudonyms.

**Table.** Participant Characteristics of Selected Transcripts Included in the Secondary Analysis.

Characteristic	Study 1 Interview Participants	Study 1 Follow-up Interviews^a^	Study 2 Interview Participants	Total Participants
Total participants	43	19	25	68
Sex				
Male	27	11	11	38
Female	16	8	14	30
Age group				
<29	14	10	–	14
30–39	10	2	2	12
40–49	12	4	6	18
50–59	2	0	6	8
60+	5	3	11	16
Research site				
In-hours urgent care^b^	22	13	NA	22
Out-of-hours urgent care	13	5	NA	13
Dental hospital urgent care	8	1	NA	8
General dental practice^c^	NA	NA	25	25

NA, Not applicable.

a Follow-up interviews were repeat interviews conducted with participants who also participated in study 1 interviews.

b Urgent dental care provided in general dental practices with a specific contract for urgent care services in their area, in addition to their routine care services.

c Routine dental care provided in general dental practice that includes urgent care as part of routine services (not specific to the area).

We identified 4 types of dental visiting behaviors, which probably represent positions along a spectrum of patients’ orientations determined both by how much value they place on anticipatory visits to look after their oral health, intersecting with how accepting they are of dental team recommendations and perspectives (Fig.).

**Figure.**
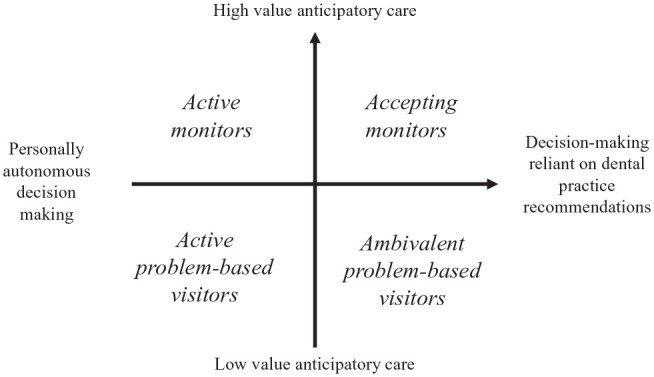
Typology of dental visiting orientations.

Each of the 4 types of dental visiting typologies is now described in turn, followed by describing individuals’ shifts between different dental visiting types.

### Accepting Monitors

Many participants described dental visits at steady intervals, often maintained in a pattern for many years. This group was highly accepting of practice recommendations in a largely uncritical way. Participants discussed visiting the dentist at such regular intervals as mainly to monitor and maintain oral health, which was in part self-directed (motivated by a principle of checking things over and catching things early), albeit also reinforced by dental service actions such as delivery of text reminders.

INT: Do you go regular, or do you go when your teeth hurt?

R: I go regularly.

INT: Is that something you’ve always done?

R: Yes, yeah . . . probably when it was my own choice if you see what I mean . . . I can’t remember much beyond as a teenager.

INT: What makes you go regularly?

R: A text that reminds me that I’ve got to go [laughs].

INT: And what makes you be the good person that says “Oh I’ll go to my appointment now then?”

R: I just think it’s appropriate . . . preventative, better than anything else. Should be cheaper if you get there first . . . before anything goes wrong. And I think it’s also something you can’t really check yourself. (Study 2, June, woman, age 60–69)

We found that reasons for dental visiting often revolved around perceiving an expectation to visit at regular intervals. This norm was often a generalized social norm, as when asked, most participants did not identify who or what gave rise to this idea, although some identified family influences. Furthermore, when participants were asked how or when they decided about this frequency of dental visits, these appeared to be often fairly passive decisions, frequently described as following the dental practice’s policies. Ken, for example, visited the dental practice he now attends following a previous dentist’s advice and goes back at intervals advised by the dentist.

INT: How long have you been going [to the dentist and the dental hygienist] every 3 months?

R: Since I started [at dental practice 2]. Roughly 4 years ago, I don’t know.

INT: Is that about the right time . . . right frequency for you? Are you happy with that?

R: Yes I think so, yes.

INT: Do they feel any different or do you feel the benefit when you’ve had them cleaned?

R: Yes, I believe they are. I believe in keeping a regular check-up, yes.

INT: Okay. Is there anything that they’ve ever advised or anything that you’ve thought . . . you’ve just thought “Oh sod off,” you know. Some people just don’t like being told about certain things. About the way they do stuff. Is there anything like that?

R: No, no I’ve always been . . . if it’s a good plumber telling me something I listen to them yes. (Study 2, Ken, man, age 60–69)

A symbiosis between the patients’ beliefs and advice received from the dental service is found. Ken described following and not actively challenging or questioning the intervals set by the dentist, for reasons of believing in keeping check-ups with regularity as well as welcoming getting recommendations from a good dentist. Thus, adopting a pattern of monitoring dental visits appears to be a consequence of both the patient’s internal motivation, which is reinforced by recommendations of the dentist with which they comply. Although in part the frequency of dental visits appears to be a fairly passive patient behavior, there were still very active patient behaviors involved in terms of fitting dental appointments around other priorities, and many participants traveled to continue visiting the same practice.

Adhering to local dental practices’ policies around dental visiting was described as shaping the patients’ pattern of monitoring visiting, even with awareness of some wider media messaging that contradicted this.

You read some of the articles and of course what you read in the press is not necessarily true, but [dentists] seem to do very well don’t they. . . . As I say, I now go every 3 months and certainly looking at some of the articles that I’ve read that’s probably over the top. I mean I probably don’t need to see them every 3 months, now I’m quite happy to go along with that as long, as it works you know. (Study 2, Dave, man, age 60–69)

Dave described his motivation to continue following the dental practice’s visiting frequency policy, despite thinking that it might not be necessary, as due to habit and having experienced trouble-free oral health: “You just get into a routine don’t you and that’s what we’ve always done so I’ve gone along with that you know because I suppose, if you think about it, it’s worked for me.”

While participants felt an obligation to visit the dentist, at the same time, there was an expectation of reciprocity, which included a number of expectations of the dentist and dental practice. First, some expected the dentist to have responsibility for ensuring their oral health:

I have had problems with my gums and one of the things that was quite sort of like shocking when [a new dentist] came on board . . . the first time I’d seen the [new] dentist they said that “Oh I’d got a problem with my gums and that potential gingivitis” or whatever. And I said “Oh hang on a minute, No, No, I’ve been coming here for 40 odd years, I’ve done everything you’ve told me to do, I’ve never missed a dental appointment ever; how come now I’ve got. . . ?” . . . It really frightened me that. (Study 2, Judith, woman, age 50–59)

Dental visiting, then, can be seen as a means of insurance against dental problems.

Second, dental practice policies around providing appointments were often discussed in the context of being limited to patients who have maintained their visiting routine.

We are lucky it’s NHS, we’re very lucky. And that’s why, that’s one of the things that keeps you wanting to go for your appointments. Because you don’t want to lapse from that. . . . Because you’d just lose your place. You’d never get back in. (Study 2, John, man, age 50–59)

Thus, the dental visiting policy by the practice was often a motivation to continue visits at intervals expected by the dental practice. Many participants compared their own experiences with those of others who had not visited a dentist for some time and did not find an NHS dentist when they wanted to start attending as a cautionary tale.

Many participants, then, were accepting of or strongly influenced by dental practice policies around dental visiting, driven by a social norm to visit the dentist for monitoring their oral health, an expectation of dental visiting for monitoring insuring against dental problems, and fear of exclusion from the practice if they did not adhere to practice recommended visiting routines.

### Active Monitors

Some participants visited the dentist out of a conscious belief in the importance of regularly monitoring their oral health. Their dental visits still followed a monitoring cycle, but rather than following the dental practice’s recall intervals, participants actively decided on the timing of dental visits themselves.

I think they are essential luxuries. But I think if it was that or something more fundamental that I needed, you know pay a bill or something to do with food, I don’t know, keeping the car on the road, you know something like that then maybe you’d try and skip going to the dentist every . . . go less often. We were going every 3 months . . . we were thinking well this is costing a bit you know. . . . Your eyes . . . you’ve got your eyes, you pay for all of that as well haven’t you. . . . My teeth are more important than my eyes you know. (Study 2, Bridget, woman, age 60–69)

Motivation to visit for monitoring, here, is then less driven by a norm or familiarity with dental visiting and more by the importance and priority oral health has to Bridget. Participants’ socioeconomic circumstances are important here too, with visiting dependent on income, which might not be the case for participants with higher income levels.

Participants were influenced by the dental practice’s policies but actively challenged or asked questions about their dental visits.

INT: Were you going for cleaning with the hygienist as well?

R: Yes, [the dentist] told me it might be beneficial to go every 3 months to have my teeth cleaned, which I was doing for 12 months. Oh well, I still do it now because he said to me “You’ve probably no need to come every 3 months now because you’ve got the situation under control.” “Your gums are a lot happier.” I said, “Well if it’s alright with you, I’d like to still come every 3 months.” To keep my teeth and gums looking good. He said, “Right that’s fine.” (Study 2, Angela, woman, age 50–59)

Above we see that the dentist is reported to have tried to adjust the number of dental visits but found Angela disagreed and wanted to be seen more often, in part to maintain a good appearance of her mouth. Indeed, these data suggest that active monitors switched dentists if they did not feel comfortable more than the accepting monitors group. A strong trust relationship with a dentist was often an important consideration in keeping visiting a particular practice.

That’s what you build on, it’s the relationship that you make that makes people want to go back to the dentist, it’s not that awful place that you know you’re messed about in . . . I book it so much in advance so I can make sure [I see the same dentist] and I then though, plan my working week around my dentist appointment. (Study 2, Tara, woman, age 30–39)

In this group, too, some participants expected a service-oriented approach by the practice. Thus, an active monitoring orientation entailed a more transactional approach, associated with more switching between dental practices, expectations of receiving a high level of service, and active decision-making on the part of the participants.

### Ambivalent Problem–Based Visitors

A substantial group of participants visited a dentist for help with problems that were urgent and did not go away by themselves, such as when in pain, having an infection, or swelling in the mouth, rather than for preempting problems by checking their oral health.

I think I’ve got an abscess on the front of me mouth here, so I never got much sleep last night. . . . Obviously through the day it’s got no better with painkillers so that’s when I phoned the emergency. (Study 1, Esther, woman, age 40–49, in-hours)

[Before visiting urgent dental care] teeth were cracking and they were starting to go discoloured and black looking and basically you know I’m like, I can’t carry on like this. . . . But, I let it get to the point where, erm, you know, it was like more teeth were getting bad. It weren’t like the first sign I noticed, like say one tooth. (Study 1, Esther, follow-up interview)

Problem-based visiting was often described by participants as starting from a nonconscious decision, where this has been relegated to a lower priority in their lives, especially where there were less resources due to challenging job demands or family circumstances. As Tom described,

I’ve probably, before I left home [going to the dentist] was like once a year. And I did have brace work done before then, when I was still under 16 so it was all free and that kind of thing. And then once I moved out and it was just, you know, a hassle to line up an appointment when you’ve also got to go to work. . . . For all I know I’ll go in tomorrow and me shift for Friday will have changed so it’s kind of hard. I’ve kind of neglected dentists and, well, any kind of appointment, doctor included. (Study 1, Tom, man, age 30–39, in-hours)

Dental visits reverted to problem-based visiting by default. In Tom’s account, monitoring visits still feature as a social norm that is “neglected,” yet one that he was prepared to ignore. This shift could be characterized as an ambivalent attitude, leading to a fairly passive decision of moving to problem-based visits without actively rejecting monitoring visits or deciding when to visit.

Over time, an orientation toward problem-based visiting became increasingly pronounced. Some participants, for example, described initially not visiting a dentist due to not having a need, but this then combined with dental practice policies that made it difficult to return. Thus, service-related factors that favored “regular” attenders meant that those who had a lower priority for anticipatory care in their lives were more likely to become “problem based” by default.

R: Er, about 5 years ago in [another region], I think that was the last time

INT: Was there any reason why you didn’t go back to a dentist after that?

R: Erm, I just never had a need, like I never had toothache, and I think the previous dentist that I went to, after you don’t go there for 2 years, they forget you were a patient. So, I had that problem, so I never bothered to. . . . It was like going the doctor’s, I don’t go the doctor’s unless I need to go the doctor’s. (Study 1, Lewis, man, age 20–29, in-hours)

Lewis’s orientation to dental visits, then, became fully problem based in the period he describes, identifying it with curative visits only, in an analogy to primary medical care visits.

Dental practice policies, however, were experienced quite differently from primary medical practice policies. Many participants, for example, described attempting to make an appointment for an urgent dental problem at a practice they had previously visited and being told that due to not visiting for some years, they would need to wait to be seen as a “new patient.” Such experiences, or expectations based on similar experiences, could become a further reason not to attempt to make any appointments.

I haven’t got a dentist. Saying that, about 5 years ago I come here [at the dental practice where he attends for urgent care] to get another tooth pulled. And I ended up being on the books, having this as me local dentists. But, not receiving any follow-ups like come in get a check-up and all that, I just haven’t bothered with the dentist. . . . But with not receiving any check-ups, like you know you have to go for a check-up I automatically thought it’s run out, run its course, finished. (Study 1, Lee, man, age 60–69, in-hours)

In a follow-up interview a few months after this interview, when he attended for an urgent care appointment, Lee discussed that he reattended at this practice and wanted to remain “on their books” now.

The concept of registration or “being on the books” lapsing after some time was often discussed. Although registration with a doctor is part of the health system for primary medical care in the United Kingdom, it is not currently part of the system in English general dental practice (it has been previously before the NHS dental contract was revised in 2006). While legally, general dental practices only have an obligation to care and take on patients who are under active courses of treatment, in practice, because of an institutional logic of autonomy geared toward sustainability of the practice as a business enterprise that is dependent on committed and regular users, dental teams often prioritize “regular” patients (Harris and Holt 2013).

Getting a dentist appointment when not being on a recall list could become more of an issue where dental practices’ capacity is limited. Those who are not prioritized and are not under active courses of treatment can access urgent care in the United Kingdom under a regionally managed system that triages appointments for urgent care patients and directs them to a limited number of urgent care settings, with a set number of appointments available throughout the region each day. This service provides a system whereby patients can get care for their urgent problem, but once this has been addressed, they would still be without a dental practice “home” for any ongoing care or to address other disease in their mouth that is not yet causing problems. Urgent dental care appointments could then be the only resort, as the partner of this participant stated, comparing her own experience with visiting a practice at regular intervals for monitoring with the experience of her partner, who had not visited a dentist for some years.

Over a year ago I had a little bit of a tooth pain, I called up [the dental practice where she goes for check-ups at regular intervals and treatment] and they saw me the same day. ’Cause they do emergency appointments for the patients, so, they’re really good. . . . When your teeth are ok I think you can forget how awful the pain can be. And I think the preventative treatment is better than that worry of suddenly needing to see a dentist in an emergency. Because it is hard, I think if you’re not registered with a dentist. It’s a little bit of a nightmare, because there’s nothing you can do, you know, if you’re really ill you can go to A&E, or things like that, whereas dental pain is so bad, but it’s a little bit, there’s nothing you can do. It’s a case of hoping to get an emergency appointment or nothing. . . . It just reminds you of the importance of being with a dentist. . . . [Otherwise you have] that worry of not being able to get seen quick enough. (Study 1, interview with Grace, interviewee woman, aged 30–39, accompanying partner Mark to urgent dental care appointment in-hours)

### Active Problem–Based Visitors

Problem-based dental visiting was an active decision for some participants, who engaged with dental visiting only when needing treatment.

R: See I’ve had 55 addresses. I’ve moved around too much. I don’t even have a regular GP. And because of my job I only ever stay in one place for 3 years tops and then I have to move [unclear]. So it’s just not having the time. Not only do I have to make an appointment I then have to find somewhere first. And it’s the whole rigmarole of doing it. . . .

INT: So is there anything that would make you go to a dentist before you were in pain actually?

R: Probably not. To be honest, probably not. I’m meant to go to asthma check-ups things like that. I’ve never been for an asthma check-up. If I had children, it’d be different because then you would be leading example. I have no kids. I’m just one of those people, unless I’m in pain. . . . (Study 1, John, man, age 40–49, in-hours)

Here, it is John who decides when to visit for a dental appointment, not the dental practice’s expectations. John discussed in his interview that he kept up his self-care at home and engaged with dental treatment when he felt the need for treatment was high and surpassed what he could take care of himself. His approach was not limited to dental visits only but extended to other types of health care too, which he felt were difficult to use as he had to find a new service in every place he lived, which was relevant only when taking a longer-term approach, not within his current life with short periods remaining in one place.

Dental visits here mainly occur when having a dental problem or for the duration of a course of dental treatment, for example, to complete a filling.

INT: Do you think of going to the dentist more after you get the problem sorted today?

R: No. I mean I will have to go ’cause I’ll have to get a proper filling. But after that. . . . ’Cause like I said I just, I just don’t have the time and if I don’t think there’s anything wrong with me then I’m not gonna go. . . . It’s like you’ve got to go and like sit for an hour in a dentist. And pay, pay for a check-up and then there’s nothing wrong with you. Why do you want to waste that time, and that money? If there’s nothing there. Yeah, that’s why I don’t go. (Study 1, Jenny, woman, age 30–39, in-hours)

The idea of anticipatory care dental visits was rejected by many participants in this group either as not relevant to immediate priorities or because preventive care was perceived as unnecessary. Although the urgent dental care services described above provide the problem-based appointments that active problem–based visitors mostly use, these services often recommend finding more ongoing care arrangements, which would involve a more anticipatory dental care approach.

R: This [visiting urgent dental care] is my plan B to be honest with you. My plan A and B. I’d be lost without this right now. . . . To be honest with you stupidly, I’m not registered with me own dentist. Erm, I have been on and off over the years. And I’ve owed them money over the years so I’ve been taken off the system. Then when things get too bad I need to come to an emergency dentist. They force me into a decision where then I’ve got to go and register to see a dentist. I get so far with treatment and the money puts me off. . . .

So I’d leave it until it was beyond it, ’til painkillers don’t do anything, I’ll get to that last resort then I’ll go the Dental Hospital. Erm, they’ve been brilliant [unclear] but they tell you off as soon as you go in there. First thing they say is get your own dentist. . . .

INT: How often have you been in that situation where you’ve had that much pain basically?

R: Over 10 times, minimum of 10 times. I’ve been in and out of that place over the years. (Study 1, Chris, man, age 40–49, out-of-hours)

It is apparent that a spectrum of dental visiting exists with visiting for a one-off appointment while having a dental problem at one pole and an ongoing relationship with a dental practice, involving a series of check-ups with associated courses of treatment where these are needed, at the other pole. There are also situations between these 2 arrangements, whereby the urgent care appointment prompts either a follow-up appointment to check on the success of the urgent care intervention or to fully complete the treatment for the dental problem that was begun at the first dental visit. Sometimes the patient may then go on to complete a course of treatment to address other disease in the mouth that had yet to cause symptoms. Chris described starting but not finishing treatment courses to address other dental disease. Other active problem–based visitors sometimes completed courses of treatment (oral health stabilization) but not taking up a recall appointment to monitor their oral health some months down the line.

### Turning Points in Dental Visiting

Some participants experienced turning points in their dental visiting orientation. As described above, accepting and active monitors could undergo lapses in dental visiting, with interruptions due to changes in their life conditions such as job demands or their family situation. When these lapses occurred for some time, their characteristics shifted toward a problem-based profile. Ambivalent problem–based visitors could shift toward an anticipatory care trajectory, often but not always after an urgent care visit. Many participants discussed the experience of policies around urgent dental care as a motivation to move into monitoring dental visiting and discussed registering with a dentist as a means to ensuring gaining access to care.

INT: Do you think that from now on you’ll be going a bit more regularly to the dentist than before?

R: Yeah.

INT: Why is that? What made the change?

R: Erm, it’s because I’ve got a registered dentist now.

INT: Yeah. So, why does that make you go more regularly?

R: Erm, just because I don’t have to worry about registering as a patient and worrying about getting seen to and the messing about bit. . . . I think I’m confident going to the dentist now. (Study 1, follow-up interview with Lewis, man, age 20–29, in-hours)

In Lewis’s account, taking up anticipatory care created confidence in visiting the dentist, which he further explained as a result of knowing his dental problems were now being taken care of.

Such turning points were often participant-initiated decisions but were often reinforced by social support or encountering a dentist who was trusted at these moments of change. Participants’ decisions at turning points were often linked to life conditions, such as changing family circumstances and changes in job conditions or other changes affecting their time and financial resources. Kate, for example, discussed in a follow-up interview that she decided after her urgent visit to start more regular dental visits as part of taking more care of herself after having her first child. When asked if she would continue with dental visits, she described the dentist she had an appointment with as instrumental in her motivation.

It was just me, I’m not scared of the dentist or anything like that, it was just me not thinking like, probably not looking after myself really isn’t it, because I should have been going but I just didn’t think to even book the dentist. Because I’ve never had an issue or anything like. But I definitely will now. I’d say it was the dentist [whom she had a check-up with after 2 urgent dental care visits] as well . . . she was like, “you really need to come, there’s nothing wrong with your teeth but you really need to come the dentist and get them checked.” [She] gave me a push. She was like “go downstairs and book an appointment now for a check-up,” and I was like ok. (Study 1, follow-up interview with Kate, woman, age 20–29, out-of-hours)

Participants with an active problem–based orientation could get to a turning point in their motivation, often when finding a dentist with whom they established a good relationship. Here, forming a relationship with a dental practice was part of establishing an anticipatory care orientation.

R: It was really nice because . . . it’s quite a nice surgery inside. It’s really quick, they have a TV screen and it tells you all like the different treatments that you can have there, privately as well. So, I found that quite interesting because I did want to have a couple of private dentistry done. And the staff are really nice so, I’ve quite enjoyed going to the dentist.

. . .

R: Erm, I suppose I will be going back more regularly, yeah.

INT: What makes you think that?

R: Erm, just because I’ve created a relationship with the dentist. So, I wouldn’t want to, like, let her down. (Study 1, follow-up interview with Jenny, woman, age 30–39, in-hours)

Jenny, who at the urgent dental visit discussed avoiding monitoring dental visits as unnecessary, thus described a shift in her motivations following finding a dentist who provided the treatments she felt she needed and creating a relationship with the dentist. Although she did not express certainty that she would continue with dental visits, she had started to engage with the dentist’s expectations. Thus, motivations for completing a course of treatment—when supported by a positive dentist–patient relationship—can lead to a shift from low to moderately higher valuing of anticipatory care, which may be a first step toward monitoring visiting.

## Discussion

This article explores patients’ accounts of the purpose of dental visits and how dental visiting behaviors are shaped by dental care services. We argue there are distinguishable patterns of accepting and active monitoring, as well as ambivalent and active problem–based dental visiting behavior. These patterns differ in following dentists’ recommendations about dental visiting frequency and accepting dental visiting policies to ensure oral health compared with making active decisions regarding dental visiting based on importance of oral health. Furthermore, patterns also differ between people who see the purpose of dental visiting as revolving around following social norms of monitoring oral health and maintenance (monitoring) and this purpose being neglected or rejected, attending for resolving dental problems that cannot be self-managed (problem based). Although some people’s dental visiting remains a stable, long-term pattern, others change from one pattern to another at turning points in their trajectory. Thus, dental visiting is not dichotomous (regular vs. irregular) in people’s accounts. The findings of this analysis inform health policy around supporting uptake of preventive health care visits, contributing in particular to understanding how risk-based preventive visiting policies may be better adapted to patients’ understanding of the purpose of visits, and that this is in part shaped by service-related factors in an ecological way (Harris 2016), arising from habituated practices of both patients and dental teams (Harris and Holt 2013).

Our findings show that dental visiting behaviors need to be understood as distinct patterns that go beyond a regular versus problem-based visiting dichotomy. Thus, this study supports and extends insights from longitudinal studies (Thomson et al. 2010; Aldossary et al. 2015; Broadbent et al. 2016) and further adds crucial insight into reasons for regular and irregular dental visiting (Anderson and Morgan 1992; Currie et al. 2021). We found that rather than categorizing patients into attenders and nonattenders, there are personally autonomous and dental practice recommendation-reliant types of behaviors toward dental visiting policies among both patients who visited for monitoring and among those who visited for dental problems. Moreover, these patterns can change over time, supporting the need for longitudinal perspectives on dental visiting (Thomson et al. 2010; Aldossary et al. 2015; Broadbent et al. 2016). These findings show the importance of paying attention to the interplay between health care services and patients constituting and defining the purpose and appropriateness of health care visits (Dixon-Woods et al. 2006; Harris 2016).

Patients’ understanding of the purposes of dental visiting and the links this has with dental visiting behaviors shows strong similarities with studies on preventive visiting in general medical care services. The concept of preventive visits as insurance, with the health care provider viewed as taking responsibility for maintaining health, has similarly been found in patients’ accounts of eye screening (Byrne et al. 2020). This may underlie in part patients’ preferences toward dental visits at standard, regular intervals compared with risk-based intervals (Fee et al. 2020). The use of risk-based intervals between preventive visits rather than standardized intervals is contested by patients in various areas of preventive health care visits, due in part to worries about longer intervals not being sufficient for preventing disease, cynicism about cost-saving motives on the part of health care services rather than health promotion motives, and regular visits being seen as an important motivation for self-care maintenance (He et al. 2018; Byrne et al. 2020).

Tacit understanding around “registration” acting as insurance of access to appointments and as a motivation for compliance with recommended visiting intervals is, however, particularly pertinent to dental visiting. Previous studies have identified that dental patients are often stereotyped as “good patients” or “bad patients” on the basis of their dental attendance patterns (Pegon-Machat et al. 2009; Bedos et al. 2014). As our study shows, these differences in how patients are approached by dentists, depending on their perceived dental visiting behavior, may in turn influence patients’ behaviors and increase barriers to visiting in a dynamic way, and this may be a reason why the system tends to see substantial inequalities in health (Sanders et al. 2006).

The COVID-19 pandemic has significantly affected rates of dental visiting across the whole UK population, due to a period of closure of all routine dental services between March and June 2020 and some ongoing restrictions in service capacity due to implementation of necessary new Infection, Prevention, and Control protocols (NHS Digital 2021). The lower availability of appointments may further widen the divide between those who have had a period of problem-based visiting who do not have an available dentist and those who are on a recall list having attended planned appointments who have an available dentist. At the same time, the need for prevention and oral health care particularly in populations more at risk of dental diseases is high (Brian and Weintraub 2020), which makes availability of dental care services for people with a period of problem-based visits even more pressing, before the pandemic and more so since.

The analysis presented in this article has some limitations. The data sets used focused primarily on participants experiencing changes in their oral health, and the included studies were primarily conducted in areas of socioeconomic disadvantage. More research is needed to confirm if the accounts of dental attendance found here also hold for people with stable oral health and in wealthier areas, where other priorities and access to resources may differ. Second, in both studies analyzed in this article, the interviewers used the terms “regular” visiting and visiting “when in pain” or “when having a problem,” which is in line with the pervasive view of dental visiting as dichotomous, which we challenge in our analysis. Thus, participants may have been influenced in their accounts by the interviewers’ use of these terms. However, these terms were also used by some participants without prompting. Other terms like “visiting for check-ups” or “visiting for planned dental visits” were often not understood in the same way by all participants, whereas “regular visiting” was more accepted. The main strength of this study is that it increases understanding of patients’ dental visiting patterns across 2 different settings, which increases external validity and adds to much-needed understanding of how dental visiting patterns may be shaped by dental care services and by patients’ understanding of the purpose of visiting.

In conclusion, patients’ views on the purpose of dental visits, as well as the way patients respond to dental care services’ shaping of dental visiting, shape visiting patterns. This article helps inform health policy approaches to reducing inequalities in preventive visiting to move beyond regular versus problem-based visiting categorizations. The article also suggests that population surveys should be designed with awareness of and language reflecting the various points on the spectrum of dental visiting patterns. In addition, the consequences of dental care service practices that differentiate between patients who are on a recall list for preventive visiting behaviors and those who are not should be carefully considered in light of policy ambitions to reduce health inequalities.

## Author Contributions

M.M. van der Zande, contributed to conception and design, data acquisition, analysis, and interpretation, drafted and critically revised the manuscript; C.E. Exley, R. Freeman, contributed to conception and design, data analysis and interpretation, critically revised the manuscript; C. Thetford, contributed to data acquisition, analysis and interpretation, critically revised the manuscript; R.V. Harris, contributed to conception and design, data acquisition, analysis and interpretation, drafted and critically revised the manuscript. All authors gave final approval and agree to be accountable for all aspects of the work.

## Supplemental Material

sj-docx-1-jct-10.1177_23800844221118515 – Supplemental material for Is Dichotomization into Regular versus Irregular Dental Attenders Valid? A Qualitative Analysis Click here for additional data file. Supplemental material, sj-docx-1-jct-10.1177_23800844221118515 for Is Dichotomization into Regular versus Irregular Dental Attenders Valid? A Qualitative Analysis by M.M. van der Zande, C.E. Exley, R. Freeman, C. Thetford and R.V. Harris in JDR Clinical & Translational Research
