# Supplementary material for: Is Dichotomization into Regular versus Irregular Dental Attenders Valid? A Qualitative Analysis
Source: JDR Clin Trans Res. 2022 Aug 29;8(4):337–48. doi: 10.1177/23800844221118515 (PMC10504811; doi:10.1177/23800844221118515)
Supplement: sj-docx-1-jct-10.1177_23800844221118515 – Supplemental material for Is Dichotomization into Regular versus Irregular Dental Attenders Valid? A Qualitative Analysis [file sj-docx-1-jct-10.1177_23800844221118515.docx]

Supplemental file to “Is dichotomisation into regular versus irregular dental attenders valid?- a qualitative analysis”

Marieke M. van der Zande, Catherine E. Exley, Ruth Freeman^†^, Clare Thetford, Rebecca V. Harris

Appendix Table 1.

Topics discussed in interviews and follow-up interviews in Study 1 with participants attending urgent dental care

| Topics discussed in Study 1 interviews |
| --- |
| Reasons for using urgent dental care |
| Last dental visit and dental visiting history |
| Factors inhibiting dental visiting for planned care |
| Intentions regarding dental visiting after urgent dental care |
| Help needed and plans for overcoming barriers to dental visiting |
| Any remaining difficulties after trying to overcome the barriers discussed |
| Topics discussed in Study 1 follow-up interviews |
| Experiences of and dental problems since the urgent dental care visit |
| Plans and actions towards finding a dentist since the urgent dental care visit |
| Experiences during any dental visits since the urgent dental visit |
| Changes in barriers to dental visiting |
| Intention to attend for planned dental care in future |

Appendix Table 2.

Topics discussed in biographical interviews in Study 2

| Topics discussed in Study 2 interviews |
| --- |
| Occupation, residential history, family situation |
| Dental visiting history and recent visits at the dental practice |
| Relationship with dentist and relationship with dental hygienist |
| Motivation regarding dental visits and oral hygiene behaviour |
| Awareness of dental problems and experience of receiving diagnosis of gum problems |
| Opinions on and behaviours towards dentist/dental hygienist’s dental visiting and oral hygiene advice |
| Communication with dental practice team |
| Overall care for self |

Appendix Table 3.

Reflections on researchers’

| Topic | Influence of researchers’ background and perspectives |
| --- | --- |
| Interviewers’ role in interview context | The main researchers in both studies (MZ and CT) have a strong interest in patients’ perspectives and how their social context influences their use of healthcare services, and both did not have a clinical background, which helped to distinguish their role from that of the dental settings the studies were conducted with. |
| Eliciting detailed accounts | The Study 2 interviewer (CT) had a long-standing link with the area the research was conducted in and was therefore able to elicit accounts by relating to the participants’ perspectives. |
| Eliciting detailed accounts | The main interviewer in Study 1 (MZ) had recently moved to the area from a different country at the time of data collection and was therefore able to elicit explanations about aspects of participants’ context from a more outsider position. |
| Aims of primary studies and research interests | The research aims and interviewers’ research interests in Study 1 led the researchers to be very attuned to the effects of socioeconomic inequalities in participants’ experiences with and responses to dental care services. |
| Aims of primary studies and research interests | The research aims and interviewer’s research interests in Study 2 led to being very attuned to patients’ responses to dental care services and how different elements of service delivery influenced participants’ behaviours in their accounts. |
